# Suppressive effects of simvastatin on the human acute promyelocytic leukemia NB4 cell line through the regulation of the nuclear factor-κB signaling pathway

**DOI:** 10.3892/ol.2014.2204

**Published:** 2014-05-30

**Authors:** GUOQIANG QIU, XIAOBAO XIE, ZHILIN WANG, MEI ZENG, TINGXIU JIANG, ZHILAN ZOU, LI DAI, XIAOYING HUA, WEIYING GU

**Affiliations:** 1 Laboratory of Hematology, The First People’s Hospital of Changzhou, Third Affiliated Hospital of Suzhou University, Changzhou, Jiangsu 213003, P.R. China; 2 Department of Hematology, The First People’s Hospital of Changzhou, Third Affiliated Hospital of Suzhou University, Changzhou, Jiangsu 213003, P.R. China; 3 Department of Hematology, The First People’s Hospital of Kunshan, Kunshan, Jiangsu 215300, P.R. China; 4 Department of Hematology, Fourth Affiliated Hospital of Guangxi Medical University, Liuzhou, Guangxi 545005, P.R. China

**Keywords:** simvastatin, NB4 cell, NF-κB signaling pathway, proliferation, apoptosis, PCR array

## Abstract

The present study examined the effects of simvastatin on the proliferation, apoptosis and gene expression levels involved in the nuclear factor-κB (NF-κB) signaling pathway in the human acute promyelocytic leukemia NB4 cell line by methyl thiazolyl tetrazolium assay, flow cytometry and the Human NF-κB Signaling Pathway RT^2^ Profiler™ PCR Array profiles. The results showed that simvastatin significantly inhibited proliferation and induced apoptosis of the NB4 cells in a time- and dose-dependent manner. Changes were noted in the expression levels of 56 genes involved in the NF-κB signaling pathways in the NB4 cells treated with 15 μm simvastatin at 48 h post-incubation, among which, 47 genes were downregulated and 9 were upregulated. In conclusion, simvastatin potentially inhibits the proliferation and induces the apoptosis of NB4 cells through the regulation of the expression levels of genes involved in the NF-κB signaling pathway.

## Introduction

Statins as a pharmacological inhibitor of 3-hydroxy-3-methylglutaryl-CoAreductase are widely used in the treatment of hypercholesterolemia in humans. Various statins have been shown to exert several beneficial antineoplastic properties, including antiproliferative effects on tumor cells, the inhibition of tumor growth, the induction of cell differentiation and apoptosis and the inhibition of the angiogenesis and metastasis of malignant cells, such as breast cancer, leukemia, prostate cancer and colon cancer cells (1–6). Studies that analyzed the use of atorvastatin and fluvastatin in the NB4 acute promyelocytic leukemia (APL) cell line found that the drugs are potent inducers of cell differentiation and apoptosis, establishing the fact that statins demonstrate potent antileukemic properties *in vitro* and indicating the possibility that statins in combination with all-trans retinoic acid (ATRA) could be effective in overcoming ATRA resistance in the leukemic cells (6). Nuclear factor-κB (NF-κB) as a nuclear factor is widely distributed in cells, and acts through regulating cytokines, chemotactic factors, growth factors, adhesion molecules and the gene expression of immunological receptors, and participating in cell differentiation, immunoreaction, inflammation, cell apoptosis and tumor growth *in vivo*. Therefore, inhibiting the activation of the NF-κB signal transduction pathway probably potentiated a novel therapeutic strategy to treat immune disease, inflammation and tumors (7). Inducible drug resistance is a major barrier to effective cancer therapy, and the activation of NF-κB may aid in the development of chemoresistance (8). In fact, chemotherapeutic agents can themselves activate NF-κB, resulting in the eventual resistance of the tumor cells to the therapy (8). Several studies have shown that, in UCN-01-treated cells, simvastatin suppressed the activation of NF-κB and potentiated the apoptosis induced by doxorubicin, paclitaxel and 5-fluorouracil (9), acting via a Ras farnesylation-associated mechanism to create signaling perturbations, particularly the prevention of Ras and ERK1/2 activation, culminating in the synergistic induction of cell death (10). However, the cytotoxic potency of simvastatin against NB4 cells and the changes in the NF-κB signaling pathway are not well clarified.

Therefore, the present study focused on the changes in the expression of the genes involved in the NF-κB signaling pathways in NB4 cells treated with simvastatin. The possible anti-leukemia mechanism of simvastatin is also discussed.

## Materials and methods

### Reagents

Simvastatin was obtained as a sodium salt from Merck Chemical Ltd., (Darmstadt, Germany) and dissolved in 99.5% ethanol to obtain a 1-mM stock solution kept at −20°C and later diluted in media prior to use in culture. The RNeasy^®^ MinElute™ purified kit was purchased from Qiagen Ltd. (Hilden, Germany). The 2× SuperArray PCR master mix and 96-Well RT^2^ Profiler™ PCR Array (catalog no. PAHS-058A) were purchased from SABioscience Ltd. (Qiagen Ltd.).

### Cell culture and treatment

The human promyelocytic leukemia NB4 cell line (kindly gifted by the Jiangsu Institute of Hematology, Suzhou, Jiangsu, China) was cultured in RPMI 1640 (Gibco Ltd., Paisley, UK) supplemented with 10% heat-inactivated fetal calf serum (Gibco Ltd, Invitrogen Life Technologies, Carlsbad, CA, USA), 100 U/ml penicillin and 100 μg/ml streptomycin in a humidified 5% CO_2_ atmosphere at 37°C. Exponentially growing cells were used for all experiments. Simvastatin was diluted with RPMI 1640 medium to the final concentrations of 15 μM (15SV), 10 μM (10SV) and 5 μM (5SV) for further treatment. The number of cells was determined by counting in a Burker chamber (Haimen Tianlong Experimental Equipment Factory, Haimen, China), and the final NB4 cell concentration was 2×10^5^ cells/ml. For the dose-response studies, the NB4 cells were seeded at 2×10^5^ cells/ml in 6-well plastic plates and treated with 15SV, 10SV or 5SV for a total treatment time of 72 h, taking NB4 cells without any treatment as normal controls. The cells of the different groups at 24 h, 48 h, and 72 h post-incubation were collected for further detection.

### MTT Assay

Cell proliferation was assessed using a methyl thiazolyl tetrazolium (MTT) assay. Briefly, the NB4 cells of the various groups with or without the indicated doses of simvastatin were seeded in 96-well flat-bottomed plates (100 ml/well; Falcon; Corning Inc., Corning, NY, USA) at a final concentration of 2×10^5^ cells/ml for the time indicated. At 24, 48 and 72 h post-incubation, the NB4 cells were incubated with 5 mg/ml MTT for 4 h at 37°C and then the medium was removed, the cells were solubilized in dimethyl sulfoxide and the absorbance was measured at 570 nm. All samples were run in triplicate. Background absorbance was corrected by subtracting the absorbance values from the wells with media alone (controls). The cell growth inhibition rate was calculated according to the following formula: Cell growth inhibition rate (%) = [1 - (absorbance of experimental group - absorbance of blank group) / (absorbance of negative group - absorbance of blank group)].

### Observation of morphological changes to NB4 cells

The NB4 cells (2×10^5^/ml) of the various groups were harvested at 24, 48 and 72 h post-incubation, washed once in phosphate-buffered saline (PBS), centrifuged at 500 × g on glass slides in a cytospin apparatus (Wescor Inc., Logan, UT, USA), and then fixed and subsequently stained with Wright-Giemsa solution (Nanjing, China). NB4 cell morphology was observed by microscope.

### Flow cytometric analysis of NB4 cell apoptosis

Apoptosis assays were performed using an Annexin V-fluorescein isothiocyanate (FITC) Apoptosis Detection Kit (Beyotime Institute of Biotechnology, Shanghai, China) following the manufacturer’s instructions, and early apoptosis was evaluated by cytofluorometry (FACScabilur, BD Biosciences, Franklin Lakes, NJ, USA). Following 24, 48 and 72 h of incubation, the NB4 cells of the various groups were collected and transferred to 5-ml plastic tubes, washed twice with cold PBS, stained with Annexin V-FITC and propidium iodide, and then analyzed by FACScabilur. Samples were run in duplicate with 10,000 events counted per sample. The apoptotic rate was expressed as the mean of three independent experiments.

### Human NF-κB signaling pathway detection by RT^2^ Profiler PCR Array

The untreated NB4 cells and those treated with 15SV were collected at 48 h post-incubation from three repeated experiments for further NF-κB signaling pathway detection. Total RNA was extracted from the NB4 cells using the TRIzol one-step procedure according to manufacturer’s instructions (Invitrogen Life Technologies), and RNA cleanup was then also performed according to the manufacturer’s instructions (RNeasy MinElute; Qiagen Ltd.). cDNA was converted using Superscript III reverse transcriptase. Quantitative PCR was performed according to the RT^2^ Profiler PCR Array instructions under the following conditions: 95°C for 10 min, then 95°C for 15 sec and 60°C for 1 min. The ΔCt value for each pathway-focused gene was calculated in each treatment group and the ΔΔCt method was used to analyze the data.

### Statistical methods

Statistical analyses were performed with SPSS software (version 16.0; SPSS, Inc., Chicago, IL, USA). All experiments were performed three times in each individual sample, and the results were presented as the mean value of the three. The Student’s t-test was used to compare the means between two groups and one-way analysis of variance was used to compare the means among more than two different groups. P<0.05 was considered to indicate a significant difference.

## Results

### Simvastatin inhibits NB4 cell growth

**Figure 1:**
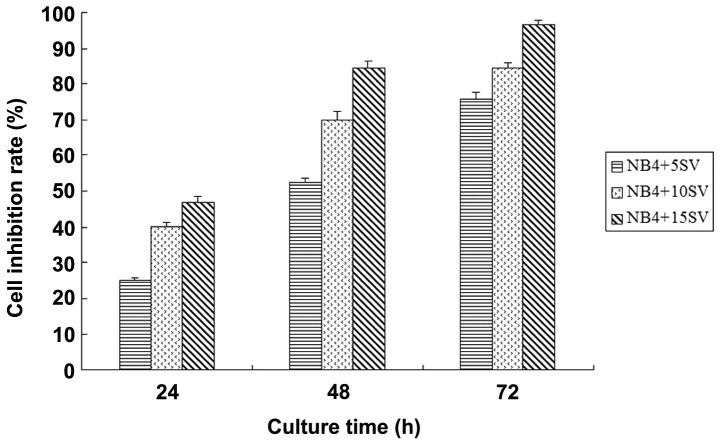
Growth inhibition rate of NB4 cells treated by varying concentrations of simvastatin at 24 h, 48 h and 72 h post-incubation. 15SV, 15 μM simvastatin; 10SV, 10 μM simvastatin; 5SV, 5 μM simvastatin.

Univariate analysis of variance of the MTT results revealed that when treated with simvastatin, the NB4 cell growth inhibition rates gradually increased with time (F=6.638, P=0.03) and dose (F=14.111, P=0.004), indicating that simvastatin potentially inhibits NB4 cell proliferation in a time and dose-dependent manner (Fig. 1).

### Morphological changes to NB4 cells treated with simvastatin

**Figure 2:**
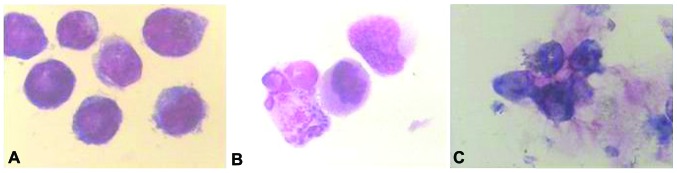
Morphological changes to the NB4 cells treated with simvastatin at the varying concentrations at 48 h post-incubation (stain, Wright-Gimsa; magnification, ×1,000). (A) Normal control NB4 cells. (B) NB4 cells treated with 10 μM simvastatin (10SV); apoptotic body formation with an increase in cytoplasm was observed. (C) NB4 cells treated with 15 μM simvastatin (15SV); evident karyorrhexis was observed by microscopy.

The NB4 cells stained by Wright-Giemsa solution exhibited karyorrhexis, petal-like nuclei and apoptotic body formation with increased cytoplasm at 24 and 48 h post-incubation when treated with simvastatin at the various concentrations. At 72 h post-incubation with simvastatin, the majority of the NB4 cells manifested karyorrhexis (Fig. 2).

### Simvastatin induces NB4 cell apoptosis in a time- and dose-dependent manner

**Figure 3:**
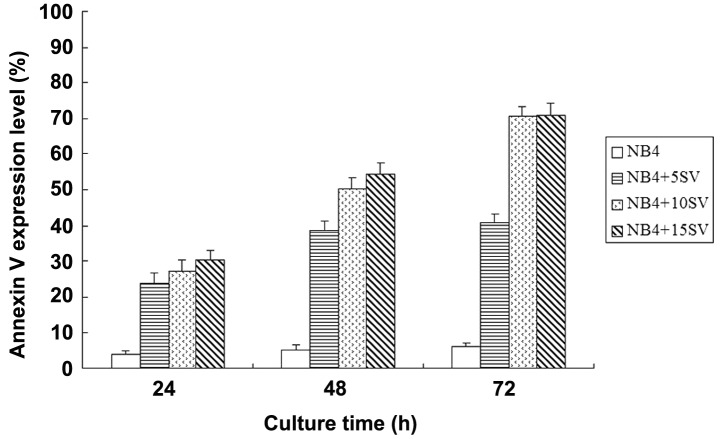
Early-stage apoptotic ratio of NB4 cells treated by the varying concentrations of simvastatin at 24 h, 48 h and 72 h post-incubation. 15SV, 15 μM simvastatin; 10SV, 10 μM simvastatin; 5SV, 5 μM simvastatin.

**Figure 4:**
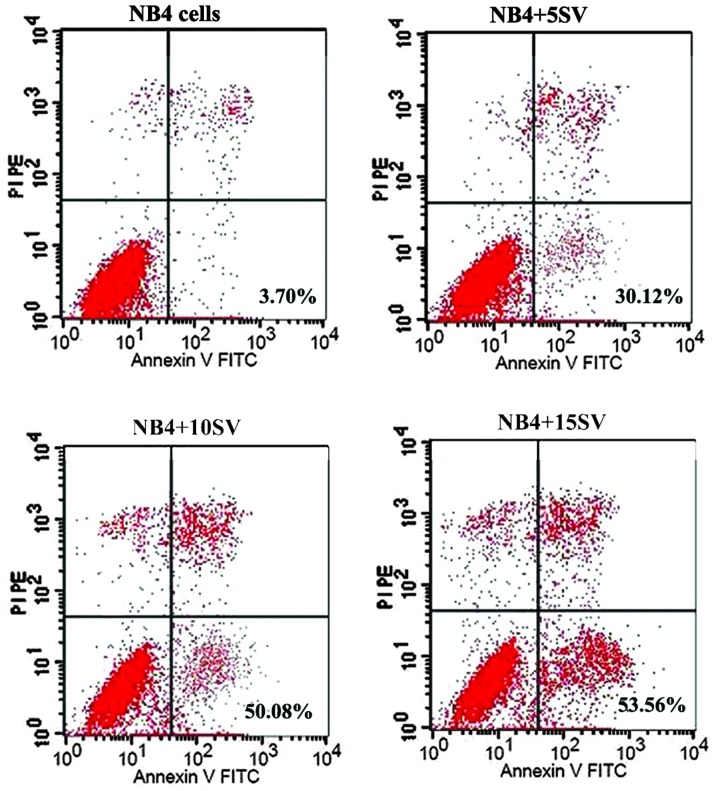
Annexin V expression levels of NB4 cells treated with simvastatin at the varying concentrations at 48 h post-incubation, as detected by FCM. 15SV, 15 μM simvastatin; 10SV, 10 μM simvastatin; 5SV, 5 μM simvastatin; FITC, fluorescein isothiocyanate; PIPE, propidine iodide/phycoerythrin.

When treated with simvastatin, the Annexin V expression levels of the NB4 cells increased in a time- (F=6.909, P=0.028) and dose-dependent (F=14.431, P=0.004) manner, and the 15SV group exhibited the highest level of apoptosis promotion, with the Annexin V expression levels of 70.49±2.68 and 70.72±3.43% at 48 and 72 h post-incubation respectively, indicating that simvastatin potentially promotes NB4 cell apoptosis (Figs. 3 and 4). Further t-tests showed that there were no statistical differences (P>0.05) in the Annexin V expression levels at 48 and 72 h in the 5SV group, therefore, untreated NB4 cells and those treated with 15SV were used at 48 h post-incubation for human NF-κB signaling pathway detection by RT^2^ Profiler™ PCR Array.

### Expression of NF-κB signaling pathway involves genes in NB4 cells treated with simvastatin

Table I shows the changes in the mRNA expression levels of the 84 genes involved in the NF-κB signaling pathway. Fold-change (2^−ΔΔCt^) is measured as the level of normalized gene expression (2^−ΔCt^) in the test sample divided by the level of normalized gene expression (2^−ΔCt^) in the control sample. Fold-regulation represents the fold-change results in a biologically meaningful way. When fold-change is >1, positive regulation or upregulation is indicated, and the fold-regulation is equal to the fold-change. However, when the fold-change is <1, negative regulation or downregulation is indicated, and the fold-regulation is the negative inverse of the fold-change. Table I shows that, among the 84 genes, the expression levels of 11 genes changed with a fold-difference of 1.5 to 2.0, and the expression levels of 45 genes manifested fold-change values of >2.0. Of the 56 differently-expressed genes, 9 manifested upregulation, including the inhibitory κB (IκB) family genes, BCL3, IκBα, caspase 8 and IFNβ; 47 manifested downregulation, including the IκB kinase (IKK) family genes, the NF-κB family genes, pro-inflammatory factors such as IL-1, IL-6, IL-8 and TNF, cellular adhesion molecule ICAM/LFA and the toll-like receptor (TLR) pathway, which mediated immune response-associated genes such as TLR family, MYD88 and IL-1 receptor-associated kinase (IRAK)1/2. The changes in the expression of these genes indicated that simvastatin may promote NB4 cell apoptosis by regulating the gene expression involved in TLR and NF-κB signaling pathways.

## Discussion

Atorvastatin and fluvastatin have previously been demonstrated as potent inducers of cell differentiation and apoptosis in the NB4 cell line (6). In another study, the following cytotoxic potency against HL-60 was found: Simvastatin (SV)>atorvastatin>cerivastatin>fluvastatin. Notably, the all-trans retinoic acid (ATRA)-resistant HL-60 variant, HL-60-R2, was twice as sensitive to SV compared with HL-60. These findings indicated that simvastatin exhibits the most cytotoxic potency against the ATRA-resistant HL-60 variant, which may overcome the ATRA resistance to APL cells (11). The present results showed that simvastatin inhibited NB4 cell growth and promoted cell apoptosis in a time- and dose-dependent manner, as found in the results of a previous study (11). It was also found that the expression levels of 56 genes involved in the NF-κB signaling pathways were changed in the NB4 cells treated with 15SV at 48 h post-incubation, and it was hypothesized that the proapoptotic mechanism may be associated with the changes in the gene expression levels involved in the NF-κB signaling pathway regulated by simvastatin. With regard to the underlying proapoptotic dose of simvastatin, it has previously been reported that combining tipifarnib and simvastatin at dose of 5 and 50 μM, respectively, exhibited a synergistic apoptosis effect in KG1 and TF-1 cells (12). The present study found that 15SV manifested clear anti-leukemia effects on the NB4 cells, avoiding the side-effects caused by high-dose simvastatin.

The expression of a wide range of genes that are involved in numerous processes, including the inflammatory and immune responses of the cell, cell growth and development, is regulated by the eukaryotic NF-κB transcription factor family. The involvement of NF-κB-mediated signal transduction has been indicated in the inflammatory response, autoimmune diseases, tumorigenesis, apoptosis and in the regulation of viral replication. NF-κB transcription factor activation occurs in response to a range of signals, including, pathogens, cytokines, injuries and other stressful conditions. NF-κB protein activation is strictly regulated, and inappropriate NF-κB signaling pathway activation has been associated with chronic inflammation, autoimmunity and a number of cancer types (13–15). Due to its critical role in cell survival, cell adhesion, inflammation, differentiation and cell growth, NF-κB has been indicated to be involved in carcinogenesis.

TLRs are a class of proteins that are required for the host defense against infection. TLRs play a key role in auto-immunity, and are considered to be important recognition and signal transduction receptors (16). MyD88 is a TLR domain-containing cytoplasmic protein. Evidence indicates that all of the TLRs, with the probable exception of TLR3, utilize this pathway. MyD88 interacts with IRAK-4, via their respective death domains. IRAK-4 then recruits IRAK-1 to the complex, leading to its phosphorylation and activation (17). IRAK-1 and IRAK-4 then dissociate from the complex and interact with TNF receptor-associated factor-6, which in turn recruits transforming growth factor-β-activated kinase-1 (TAK-1)-binding protein-1 (TAB-1) and TAB-2 to the complex. This leads to the phosphorylation and activation of the kinase, TAK-1 (18). TAK-1 then activates kinases upstream of p38 and JNK, and the IKK complex, leading to NF-κB activation and the induction of proinflammatory cytokine expression, including that of IL-1, IL-6, IL-12 and TNF-α. Therefore, the TLR/NF-κB signaling pathway upregulates inflammatory cytokine expression, which activates NF-κB, resulting in cell apoptosis inhibition and subsequent cell immortalization (18). The present results revealed that when treated with 15SV, the mRNA expression levels of the IKK and NF-κB family genes in NB4 cells were all downregulated, however, the expression levels of the IκB family genes, which inhibit NF-κB transcriptional activity, were upregulated, indicating that simvastatin promotes NB4 cell apoptosis through the inhibition of cell transcription mediated by NF-κB. Additionally, the present study found that the mRNA expression levels of the pro-inflammatory factors (IL-1, IL-6, IL-8 and TNF), the TLR signaling pathway-associated genes (TLR family genes, MYD88 and IRAK1/2), and the cell adhesion molecules (ICAM/LFA) were all downregulated, indicating that simvastatin also induces NB4 cell apoptosis through the inhibition of the expression of genes involved in the inflammation signal transduction pathway. This subsequently suppresses cell transcriptional activity. The activation of NF-κB can suppress cell apoptosis through regulating the gene expression of the IAPs family, the Bcl-2 family, the RAF family, JNK, FLIP and A20, however, the manner by which these proteins suppress cell apoptosis is not fully understood (8). Thus, suppression of NF-κB activation in cancer cells, and the subsequent induction of cell apoptosis may provide an additional target for the treatment of immune disease, inflammation and malignant tumors.

In summary, the use of simvastatin *in vitro* inhibits human acute promyelocytic leukemia NB4 cell proliferation and induces apoptosis in a time- and dose-dependent manner. The mechanism behind this may be associated with the regulation of the expression of genes involved in the TLR-mediated inflammatory response and NF-κB signaling pathways.

## Figures and Tables

**Table I:** Differentially-expressed genes involved in the NF-κB signaling pathway of the NB4 cells treated with 15SV at 48h post-incubation.

Gene	Fold-change of up- or downregulation
AGT	−7.19087
AKT1	−1.34598
ATF1	1.932655
BCL10	1.015843
BCL3^a^	3.1638
CFB	1.611881
BIRC2	1.168396
NOD1	1.352533
CASP1^a^	−2.34227
CASP8^a^	2.42342
CCL2	−19.4145
CD40	−1.00316
CFLAR	−1.08346
CHUK	−1.51554
CSF2	1.222123
CSF3	−3.36992
SLC44A2	−2.71589
EDARADD	−6.09493
LPAR1	−6.09493
EGR1	−6.70121
ELK1	−1.05755
F2R	−3.32772
FADD^a^	−1.12544
FASLG^a^	−6.09493
FOS	−6.4125
GJA1	1.139515
HMOX1	−25.0176
HTR2B	1.526521
ICAM1^a^	−87.9626
IFNA1	1.179248
IFNB1	2.015843
IFNG	−6.09493
IKBKB^a^	1.196008
IKBKE^a^	−1.52768
IKBKG^a^	−1.12064
IL10^a^	−12.9526
IL1A^a^	−3.32951
IL1B^a^	−72.6281
IL1R1^a^	−8.26226
IL6^a^	−1.72447
IL8^a^	−57.0666
IRAK1^a^	1.346327
IRAK2^a^	−31.9603
JUN^a^	−42.4192
LFA^a^	−4.13282
LTBR	1.432608
MALT1	−1.72488
MAP3K1^a^	4.217983
MYD88^a^	−2.82548
NLRP12	−40.5471
NFKB1^a^	−2.77427
NFKB2^a^	−3.9451
NFKBIA^a^	7.6537
PPM1A	1.634145
RAF1	−1.15277
REL	−1.25596
RELA^a^	−1.40901
RELB^a^	−1.72021
TRIM13	1.162488
RHOA	−2.55771
RIPK1	−1.75764
SLC20A1	2.165973
STAT1	−1.09569
TBK1	−1.2594
TICAM2	−3.75585
TLR1^a^	−3.5425
TLR2^a^	−6.09493
TLR3^a^	−1.41519
TLR4^a^	−2.27636
TLR6^a^	−6.42164
TLR7^a^	1.196063
TLR8^a^	−1.06758
TLR9^a^	−2.31402
TMED4	1.070532
TNF^a^	−22.7788
TNFAIP3^a^	−23.5035
TNFRSF10A	−1.05723
TNFRSF10B^a^	−2.53081
TNFRSF1A	2.346345
CD27	−2.32356
TNFSF10	−3.35489
TNFSF14	−1.17296
TRADD	−1.6789
TICAM1	−7.99424

a Differentially-expressed genes. A total of 9 genes manifested upregulation, including the IκB family genes, BCL3, IκBα, caspase 8 and IFNβ; 47 manifested downregulation, including the IKK family genes, the nuclear factor-κB (NF-κB) family genes, pro-inflammatory factors such as IL-1, IL-6, IL-8 and TNF, cellular adhesion molecules ICAM/LFA and the toll-like receptor (TLR) pathway, which mediated immune response-associated genes such as the TLR family, MYD88 and IRAK1/2. 15SV, 15 μM simvastatin.
